# Risks of bleeding and thrombosis in intensive care unit patients with haematological malignancies

**DOI:** 10.1186/s13613-017-0341-y

**Published:** 2017-12-11

**Authors:** Lene Russell, Lars Broksø Holst, Lars Kjeldsen, Jakob Stensballe, Anders Perner

**Affiliations:** 1 Department of Intensive Care 4131, Copenhagen University Hospital, Rigshospitalet, Blegdamsvej 9, 2100 Copenhagen, Denmark; 2 Copenhagen Academy for Medical Education and Simulation, University of Copenhagen and The Capital Region of Denmark, Copenhagen, Denmark; 3 Department of Haematology, Copenhagen University Hospital, Rigshospitalet, Copenhagen, Denmark; 4 Section for Transfusion Medicine, Capital Region Blood Bank, Copenhagen University Hospital, Rigshospitalet, Copenhagen, Denmark; 5 Department of Anaesthesia, Centre of Head and Orthopaedics, Copenhagen University Hospital, Rigshospitalet, Copenhagen, Denmark

**Keywords:** Bleeding, ICU, Intensive care, Haematology, Leukaemia, Myelodysplastic syndrome, Thrombosis, Sepsis, Transfusion

## Abstract

**Background:**

Patients with malignant haematological disease and especially those who require intensive care have an increased risk of bleeding and thrombosis, but none of these data were obtained in ICU patients only. We assessed the incidence of bleeding and thrombotic complications, use of blood products and risk factors for bleeding in an adult population of ICU patients with haematological malignancies.

**Methods:**

We screened all patients with acute leukaemia and myelodysplastic syndrome admitted to a university hospital ICU during 2008–2012. Bleeding in ICU was scored according to the WHO grading system, and risk factors were evaluated using unadjusted and adjusted analyses.

**Results:**

In total, 116 of 129 ICU patients were included; their median length of stay was 7 (IQR 2–16) days. Of these, 66 patients (57%) had at least one bleeding episode in ICU; they bled for 3 (2–6) days and most often from lower and upper airways and upper GI tract. Thirty-nine (59%) of the 66 patients had severe or debilitating (WHO grade 3 or 4) bleeding. The median platelet count on the day of grade 3 or 4 bleeding was 23 × 10^9^ per litre (IQR 13–39). Nine patients (8%) died in ICU following a bleeding episode; five of these had intra-cerebral haemorrhage. Platelet count on admission was associated with subsequent bleeding (adjusted odds ratio 1.18 (95% CI 1.03–1.35) for every 10 × 10^9^ per litre drop in platelet count, *p* = 0.016). Eleven of the 116 patients (9%) developed a clinically significant thrombosis in ICU, which was the cause of death in four patients. The median platelet count was 20 × 10^9^ per litre (15–48) at the time of thrombosis. The patients received a median of 6 units of red blood cells, 1 unit of fresh frozen plasma and 8 units of platelet concentrates in ICU.

**Conclusions:**

Severe and debilitating bleeding complications were frequent in our ICU patients with haematological malignancies, but thrombosis also occurred in spite of low platelet counts. Platelet count on ICU admission was associated with subsequent bleeding.

**Electronic supplementary material:**

The online version of this article (10.1186/s13613-017-0341-y) contains supplementary material, which is available to authorized users.

## Background

Acute leukaemia and myelodysplastic syndrome are devastating diseases that, in worst cases, involve life-threatening complications such as bleeding, sepsis, respiratory failure and renal failure, often caused by disease or treatment-related pancytopenia [1–4]. Some patients will require admission to the intensive care unit (ICU), and although the mortality for these patients appears to have declined over the last two decades, it continues to be very high [4–6].

Patients with malignant haematological disease and especially those who require intensive care have an increased risk of bleeding and thrombosis [7–11], but none of the studies were in ICU patients only. The prevention of haemorrhage and thrombosis is a challenging task as the risk of both complications is likely to increase in patients with sepsis [12–14], which is very frequent in ICU patients with malignant haematological disease [2, 5]. Thrombocytopenia increases risk of bleeding complications [11], and platelet transfusions remain the cornerstone in treatment and prevention of bleeding [15, 16]. Large multicentre studies on prophylactic platelet transfusions in thrombocytopenic non-ICU patients have shown that a restrictive prophylactic platelet transfusion strategy is likely to be safe [9], although the degree of restrictiveness is still controversial [10]. The vast majority of patients with malignant haematological disease are receiving blood products [17] so complications secondary to transfusions are also likely [14].

Because of these complexities and the lack of ICU data, we aimed to assess the following: incidence of bleeding, time to onset, risk factors for bleeding, transfusion requirements, risk of thrombotic complications and association with death in an ICU population of patients with haematological malignancies.

## Methods

All patients with acute myeloid leukaemia (AML), acute lymphoblastic leukaemia (ALL) and myelodysplastic syndrome (MDS) admitted to the general ICU at Copenhagen University Hospital, Rigshospitalet, between 1 January 2008 and 3 December 2012 were identified through the local electronic ICU database (CIS, Daintel, Copenhagen). All electronic files including computed tomography (CT) reports, ultrasound scan reports and autopsy reports, when available, were reviewed. Use of pro-coagulant and anti-thrombotic medications was collected from the electronic medication charges and blood transfusion usage from the Blood Bank database.

Bleeding and thrombosis data were reviewed every single day of the ICU admission. The electronic patient chart system has a pre-defined section for coagulation issues, prompting the physician to prospectively fill out data on coagulation/bleeding/thrombosis status on a daily basis. Bleeding was graded retrospectively according to the WHO criteria [18]. The WHO grading system, which is the most common method for categorising bleeding severity in platelet transfusion trials [10, 19, 20], categorises bleeding episodes as grade 1 (mild), grade 2 (moderate), grade 3 (severe, requiring red blood cell (RBC) transfusion within 24 h) or grade 4 (debilitating or life-threatening). Details of grading system are presented in Additional file 1: Table S1.

The blood transfusion products used were standard pre-storage leuko-reduced RBC suspended in saline–adenine–glucose and mannitol, fresh frozen separated donor plasma (with 70% of coagulation factors preserved) and platelet concentrates from four donors. All transfusions were type/cross-match compatible. One unit RBC = 245 ml, one unit fresh frozen plasma (FFP) = 275 ml, and one unit platelet concentrates = 350 ml.

Daily platelet counts were done with an automated haematology analyser (Sysmex XE-500, Denmark). Thrombocytopenic patients with bleeding had platelet counts analysed twice a day. Platelet counts of less than 10 × 10^9^ per litre were manually counted.

Simplified Acute Physiology Score (SAPS) II and the Sequential Organ Failure Assessment (SOFA) score were calculated using the worst value for that variable during the first 24 h of ICU admission [21, 22].

### Statistical analysis

Qualitative data were analysed using Mann–Whitney test for nonparametric data. For categorical data, the Chi-square test was used, and in the few cases when expected values were less than 5, Fisher’s exact test was used. The odds ratio (OR) was calculated where appropriate and expressed together with 95% confidence interval (CI). Risk factors for bleeding with a *p* value of less than 0.10 in unadjusted analyses were included in a multiple logistic regression analysis. Survival analysis of bleeding within the first 3 days in the ICU was conducted using the Kaplan–Meier method, and log-rank test was used to compare the survival curves. The linear assumptions for qualitative data included in the regression models were tested with linear splines with knot points at the quartiles. We used SAS version 9.4 (USA) and GraphPad Prism 6.00 for OS (USA) for the analyses and considered any differences statistically significant if the two-sided *p* value was less than 0.05.

## Results

### Clinical and general characteristics

One hundred and twenty-nine consecutive patients with ALL, AML or MDS were admitted during 2008–2012 and screened for inclusion; 13 patients were excluded from the study due to post-procedural observation (*n* = 11) and observation less than 24-h due to allergic reactions (*n* = 2). Thus, 116 patients were included (Table 1).

**Table 1:** Characteristics of the study population (*N* = 116)

Baseline characteristics		
Age	60	(48–66)
Female	52	(45)
Diagnosis		
ALL	15	(13)
AML	75	(65)
MDS	26	(22)
Time from leukaemia diagnosis to ICU admission		
Less than 3 months	35	(30)
3–6 months	12	(10)
6–12 months	20	(17)
More than 12 months	50	(43)
Chemotherapy within 6 weeks prior to admission	76	(66)
Haematopoietic Stem Cell Transplantation (HSCT)	43	(37)
Graft-versus-host reaction	20	(17)
Relapse after having received treatment	20	(17)
Transformation from other haematological malignancy	18	(16)
From MDS to AML	10	(9)
From CML to AML	2	(2)
From CMML to AML	2	(2)
From Mb. Waldenstrom to AML	1	(1)
Respiratory or systemic fungal infection	19	(16)
Primary reason for ICU admission		
Respiratory failure	59	(51)
Septic shock/severe sepsis	39	(34)
Bleeding	3	(3)
Severe graft-versus-host reaction	3	(3)
Other	13	(11)
WBC count at admission (× 10^9^ per litre)	3	(0.2–11)
ICU characteristics		
Sepsis^a^	109	(94)
Sepsis	9	(8)
Severe sepsis	18	(15)
Septic shock	82	(71)
Mechanical ventilation in ICU	101	(87)
Vasopressor in ICU	95	(82)
Renal replacement therapy in ICU	42	(37)
Emergency surgery immediately before or during ICU stay	3	(3)
SAPS II	58	(50–75)
SOFA (admission)	12	(9–14)
SOFA (admission) without platelet score^b^	8	(6–11)
SOFA (maximum)	14	(11–17)
Length of ICU stay, days	7	(2–16)

Values are number (%) or median (IQR)

*AML* acute myeloid leukaemia, *ALL* acute lymphoblastic leukaemia, *MDS* myelodysplastic syndrome, *CML* chronic myeloid leukaemia, *CMML* chronic myelomonocytic leukaemia, *SAPS II* Simplified Acute Physiology Score, *SOFA* Sequential Organ Failure Assessment score, *WBC* white blood cell

^a^According to the International Sepsis Definitions 2003 [23]

^b^SOFA score at admission minus score values given for platelets count (× 10^9^ per litre): ≥ 150: score = 0; ≥ 150: score = 0; < 150: score = 1; < 100: score = 2; < 50: score = 3; < 20: score = 4

The primary reasons for ICU admission were respiratory failure and severe sepsis/septic shock; 109 (94%) of the patients fulfilled the criteria for sepsis, severe sepsis and septic shock according to the International Sepsis Definitions of 2003 [23], and 82 patients (71%) had shock on admission. The median admission SAPS II and SOFA scores were 58 (interquartile range (IQR) 50–75) and 12 (9–14), respectively.

### Bleeding and platelets

In total, 66 patients (57%) had one or more bleeding episodes during the ICU stay (WHO grade 1–4). The median number of days with bleeding was 3 (IQR 2–6). One-third (*n* = 38, 33%) of the patients had a bleeding episode during the first 24 h in ICU, and 49 patients (42%) had an episode during the first 5 days. The 116 included patients spent a total of 1728 days in the ICU and of these, 333 (19%) were days when bleeding occurred.

The majority of the patients bled from more than one location; only 17/66 (26%) bled from one location only (Table 2). The most common locations were lower/upper airways and upper gastrointestinal (GI) tract. Respiratory tract bleedings were more common among mechanically ventilated patients [49/101 (49%) vs. 1/15 (7%)]; however, three patients were intubated and mechanically ventilated due to bleedings in the airways. Six patients developed intracranial bleeding (ICH). One of the patients with ICH had severe leukocytosis (WBC > 100 × 10^9^ per litre) with predominance of myeloblasts, known to be associated with increased risk of intracranial haemorrhage [24].

**Table 2:** Anatomical site of bleeding

Intracranial bleeding	6	(5)
Upper airway bleeding^a^	23	(20)
Lower airway bleeding	41	(35)
Upper gastrointestinal bleeding	22	(19)
Lower gastrointestinal bleeding	14	(12)
Skin bleedings^b^	17	(15)
Bleeding from sites of vascular catheters	13	(11)
Urinary tract	10	(9)
Eyes^c^	1	(1)
Post-biopsy^d^	4	(3)
Post-surgical bleeding	3	(3)
Eyes^c^	1	(1)
Other sites^e^	7	(6)

All values are number (%)

Many patients bled from more than one site: 17 (26%) bled from one site only; 25 (38%) bled from two sites; 12 (18%) bled from three sites; 12 (18%) bled from four or more sites

^a^Upper airway bleeding included bleedings from the nose, mouth, pharynx and larynx

^b^Skin bleedings included petechia, purpura and ecchymosis

^c^Subconjunctival bleeding

^d^The biopsy sites were: liver (*N* = 2), skin and bone marrow (pelvis)

^e^Other sites included vaginal (non-menstrual) bleedings, pelvic bleedings, a pericardial bleedings and a bleeding from spontaneous spleen rupture

**Fig. 1:**
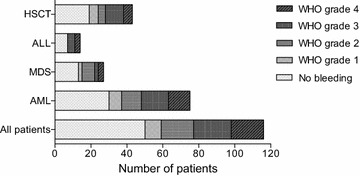
Bleeding complications during ICU stay. No significant difference was seen in number of bleedings complications between the different diagnoses or in patients with haematopoietic stem cell transplantation (HSCT). *ALL* acute lymphoblastic leukaemia, *MDS* myelodysplastic syndrome, *AML* acute myeloid leukaemia

More than half of the patients with bleeding had a severe (WHO grade 3) or debilitating (WHO grade 4) bleeding episode (39 patients, 59%). There was no significant difference in the number of bleeding complications when stratifying for ALL, AML and MDS patients (*p* = 0.67), and haematopoietic stem cell transplantation (HSCT) patients had similar numbers of bleeding complications to non-HSCT patients (*p* = 0.86) (Fig. 1).

Patients’ median platelet count at ICU admission was 29 × 10^9^ per litre (IQR 18–48), and the majority (88 patients, 76%) were severely thrombocytopenic at admission with platelet counts < 50 × 10^9^ per litre.

**Fig. 2:**
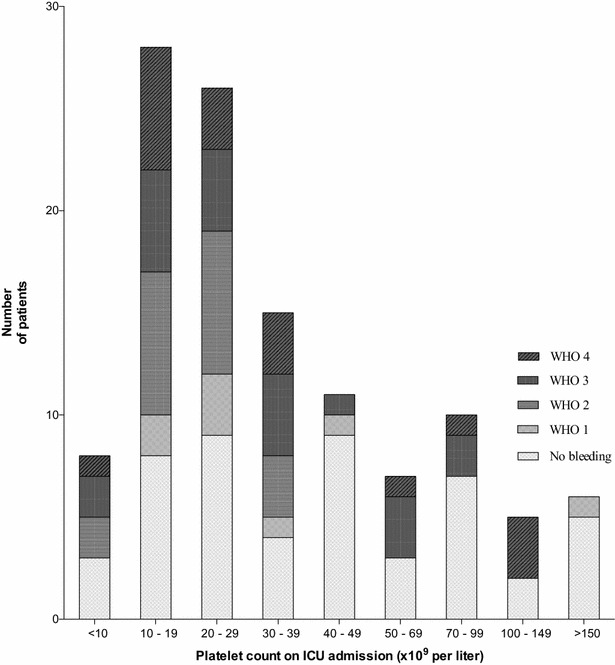
Bleeding complications at different platelet levels. This figure shows the range of platelet level at ICU admission across our patient population and frequency of bleeding complications in ICU graded according to WHO, in which bleeding episodes are categorised as grade 1 (mild), grade 2 (moderate), grade 3 (severe; requiring red blood cell (RBC) transfusion within 24-h) or grade 4 (debilitating or life-threatening)

The platelet count on admission was the only variable associated with subsequent bleeding (Fig. 2, Table 3). The adjusted OR for bleeding within the first 5 days in the ICU was 1.15 (95% CI 1.00–1.33) for every drop in platelet count by 10 × 10^9^ per litre, *p* = 0.058 and 1.18 (1.03–1.35), *p* = 0.016 for bleeding in the ICU. The median platelet count on the first bleeding day was 21 × 10^9^ per litre (IQR 15–31), and the median platelet count on the day of having a severe or debilitating bleeding episode (WHO grade 3 or 4) was 23 × 10^9^ per litre (13–39). In patients with subsequent bleeding in the ICU, we found that the individual differences in platelet counts on the first day of bleeding compared to at admission were minor [median difference 0 (IQR 0–0), mean − 3.8 × 10^9^(min–max: − 98 − (+ 28)).].

**Table 3:** Risk factors at ICU admission for bleeding in unadjusted and adjusted analyses

Unadjusted analyses	Bleeding within 5 days of admission			Bleeding in ICU^a^		
	No. (%)	OR (95% CI)	*p* value	No. (%)	OR (95% CI)	*p* value
Acute lymphatic leukaemia	5 (36)	1.37 (0.43–4.36)	0.60	7 (50)	1.37 (0.44–4.20)	0.58
Acute myeloid leukaemia	33 (44)	0.82 (0.38–1.77)	0.82	45 (60)	0.70 (0.33–1.51)	0.36
Myelodysplastic syndrome	11 (40)	1.08 (0.45–2.60)	0.85	14 (52)	1.31 (0.55–3.10)	0.55
Recent chemotherapy^b^	39 (51)	**3.16 (1.36–7.36)**	**0.006**	49 (64)	**2.45 (1.12–5.37)**	**0.02**
HSCT	15 (35)	0.61 (0.28–1.33)	0.22	24 (56)	0.93 (0.44–2.00)	0.86
Leukopenia at admission^c^	31 (56)	**3.09 (1.43–6.64)**	**0.004**	35 (64)	1.69 (0.80–3.56)	0.16
Fungal infection^d^	10 (48)	1.31 (0.51–3.37)	0.58	14 (67)	1.65 (0.61–4.47)	0.32
Renal replacement therapy	11 (48)	1.33 (0.53–3.32)	0.54	13 (57)	0.98 (0.39–2.46)	0.97
Mechanical ventilation^e^	28 (41)	0.90 (0.43–1.90)	0.78	39 (57)	1.05 (0.50–2.21)	0.91
Use of LMWH^f^	9 (29)	0.49 (0.19–1.19)	0.11	16 (52)	0.81 (0.35–1.86)	0.62

	Median (IQR)	OR (95% CI)	*p* value	Median (IQR)	OR (95% CI)	*p* value
Age	58 (48–65)	1.00 (0.97–1.03)	0.73	59 (45–66)	1.00 (0.95–1.03)	0.97
Platelet count at admission	**22 (15–31)**	**1.16 (1.03–1.32)** ^**f**^	**0.001**	**24 (15–35)**	**1.14 (1.03–1.26)** ^**f**^	**0.004**
SOFA-score at admission	12 (9–14)	1.05 (0.96–1.15)	0.14	12(9–14)	1.03 (0.95–1.13)	0.34

Adjusted analyses^g^	Bleeding within 5 days			Bleeding in ICU		
	Median(IQR)	OR (95%CI)	*p* value	Median (IQR)	OR (95% CI)	*p* value
Recent chemotherapy	n/a	2.27 (0.93–5.56)	0.07	n/a	1.70 (0.70–3.95)	0.24
White blood cell count	2.8 (0.2–11)	1.01 (0.99–1.02)	0.39	1.2 (0.1–8.5)	1.02 (1.00–1.04)	0.07
Platelet count^h^	22 (15–31)	1.15 (1.00–1.33)	0.058	**24 (15–35)**	**1.18 (1.03–1.35)**	**0.016**

Bold indicates significant results (*p* < 0.05)

*n/a* not applicable

^a^One patient lost at 1-year follow up

^b^Chemotherapy within 6 weeks of ICU-admission

^c^Leukopenia at ICU admission defined as white blood cell (WBC) count < 2 (missing data = 1)

^d^Confirmed systemic or respiratory fungal infection

^e^Mechanical ventilation on day one in ICU

^f^Low molecular weight heparin (enoxaparin 20–40 mg daily)

^g^Logistic regression analysis with bleeding as outcome and chemotherapy, WBC count and platelet count as co-variates

^h^Platelet count OR for every decrease in platelet count by 10 × 10^9^ (at admission)

### Mortality

Within the first week in ICU, 33 of the 116 patients (28%) had died and in total 64 (55%) of the patients died in the ICU. The 30-day, 90-day and 1-year mortality was 60, 72 and 86%, respectively.

**Fig. 3:**
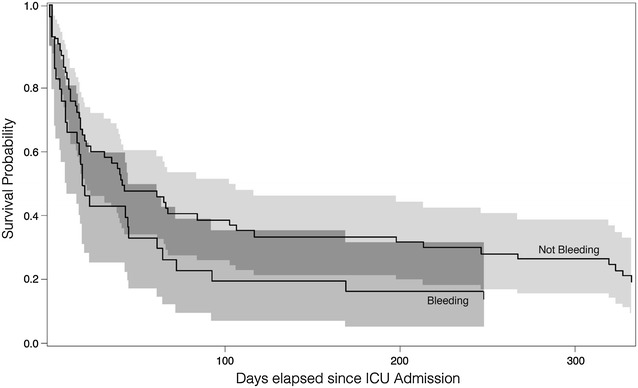
Time to death showed as Kaplan–Meier survival curves in bleeding and non-bleeding patients. The log-rank score for bleeding was 1.74 (*p* = 0.19) and the hazard ratio (HR) for patients with bleeding as compared to non-bleeders was 1.37 (95% CI 0.86–2.19). Survival curves are presented with 95% confidential intervals

Nine patients (8%) died in ICU following an acute bleeding episode; five of these had ICH, three had pulmonary bleeding and one a large post-operative bleeding after acute hemi-colectomy for bowel ischaemia. The unadjusted 30-day and 1-year mortality was higher in bleeding vs. non-bleeding patients (68 vs. 50%, *p* = 0.047 and 92 vs. 78%, *p* = 0.022), but the Kaplan–Meier survival analysis curve stratified by bleeding or not within the first 3 ICU days did not indicate any difference between the two groups (Fig. 3). Including age and SOFA score at admission in the survival analysis, the HR for death in bleeding versus non-bleeding patients was 1.28 (95% CI 0.79–2.1, *p* = 0.31).

### Transfusions

**Fig. 4:**
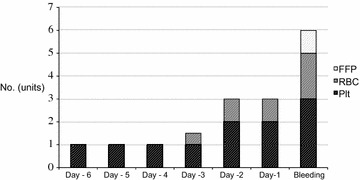
Blood transfusions given the days *before* a WHO grade 3–4 bleeding episode. This figure shows the median amount of blood products given during the days leading up to a major bleeding. *FFP* fresh frozen plasma, *RBC* red blood cells, *Plt* platelet concentrate

Due to lack of Danish national identification numbers, four patients did not have their transfusion data registered in the Blood Bank database. Of the remaining 112 patients, 100 patients (89%) received one or more transfusions of RBC, FFP or platelets concentrates during their ICU stay. The 112 patients received a total of 3561 units of blood products, and half of the units (1749 units, 49%) were given within the first week in ICU. Only four patients with bleeding did not receive any transfusions at all; two patients had large intracranial bleedings that was the immediate cause of death, and two had minor bleedings not requiring transfusions. The amount of transfusions increased on the days leading up to a grade 3 or 4 bleeding episode (Fig. 4).

#### Red blood cell transfusions

Ninety patients (80%) had at least one RBC transfusion during their ICU stay and received median 6 (IQR 1–13) units; 32/90 (36%) did not have any signs of clinical bleeding during the ICU stay.

Bleeding patients received a median of 10 (4–20) units of RBC during ICU stay as compared with 2 (0–7) units in non-bleeding patients (*p* = < 0.0001) or 0.78 unit/ICU day for bleeding patients versus 0.30 unit/ICU day for non-bleeding patients (*p* = 0.0003).

#### Fresh frozen plasma transfusions

Sixty of the 112 patients (54%) received a median 1 unit of FFP during ICU stay (IQR 0–4 units). The median amount of FFP administered to bleeding patients was 4 (0–12) units while in ICU or 0.28 units/ICU day. Twenty-seven per cent of the patients who received FFP did not have any signs of clinical bleeding during the ICU stay.

#### Platelet transfusions

Ninety of the 112 patients (80%) received a median 8 (IQR 2–23) units of platelets during ICU stay equalling a median of 2800 (700–8050) ml of platelets; 32 (36%) did not have any signs of clinical bleeding during the ICU stay. Patients with platelets < 50 × 10^9^ per litre received a median of 10 (4–28) units during ICU or 1.7 (0.8–2.3) units/day.

Bleeding patients received a median of 22 (8–39) units of platelets during ICU stay as compared with 2 (0–7) units for non-bleeding patients (*p* < 0.0001) or 1.8 unit/day for bleeding patients versus 0.5 unit/day for non-bleeding patients (*p* < 0.0001).

### Thrombosis

Eleven of the 116 patients (9%) developed a clinically significant thrombotic event (Table 4), which was the cause of death in four patients. The median platelet count was 20 × 10^9^ per litre (IQR 15 − 48 × 10^9^ per litre) at the time of thrombosis. Six of the 11 patients with thrombosis also had bleeding episodes during their ICU stay. Patients with thrombosis had the same amount of RBC and slightly more platelets/day than patients without thrombosis. Nine (82%) of the patients with thrombosis had received plasma transfusion as compared with 50/101 (50%) of the patients without thrombosis (*p* = 0.06) and those with thrombosis received more units of FFP per ICU day than patients without thrombosis (0.5 unit/day (0.1–1.5) vs. 0.02 (0–0.62) unit/day, *p* = 0.053).

**Table 4:** Thrombotic events in ICU

No. of thrombotic events—no (%)	11	(9)
Site of thrombosis		
Brain	5	
Arm, fingers, toes	2	
Liver veins	2	
Spleen (post-mortem finding)	1	
Inferior vena cava	1	
Haematological diagnosis		
AML	10	
ALL	1	
MDS	0	
Severe sepsis/septic shock—no (%)	11	(100)
Death directly related to thrombosis—no	4	
Bleeding prior to thrombotic event	3	
Bleeding after thrombotic event	3	
LMWH^a^	4	
Drugs given prior to thrombotic event		
Tranexamic acid	2	^b^
Vitamin K	2	^c^

Data are in number (%)

*AML* acute myeloid leukaemia, *ALL* acute lymphoblastic leukaemia, *MDS* myelodysplastic syndrome, *LMWH* low molecular weight heparin

^a^Enoxaparin 20–40 mg daily in ICU before thrombotic event

^b^Site of thrombi: 1. spleen (post-mortem finding); 2. thalamus (MRI finding)

^c^Site of thrombi: 1. inferior vena cava; 2. brain (multiple sites)

### Thrombosis prophylaxis

Forty-seven patients (41%) received the low molecular weight heparin (LMWH) enoxaparin in prophylactic doses during the ICU stay. There was no difference in bleeding complications between patients receiving LMWH and those who did not (*p* = 0.39). None of the patients received anti-platelet treatments when admitted to the ICU or during the ICU stay.

Among the 88 patients with platelet levels less than 50 × 10^9^ per litre on admission, 30 patients (34%) received LMWH and 58 (66%) did not. No difference in bleeding complications was seen between these groups of patients either (*p* = 0.13). Four of the 11 patients with thrombotic events had received prophylactic LMWH at the time of the event.

### Pro-coagulant drugs and vitamin K

Pro-coagulant drugs were administered to 11 patients (9%); 8 patients had vitamin K administered (7%), and 2 had so without prior bleeding. Both these patients developed large thrombi, which were the immediate cause of death. Nine patients received tranexamic acid.

## Discussion

This study confirmed that a high percentage of ICU patients with acute leukaemia and myelodysplastic syndrome are subject to bleeding. Low platelet count at ICU admission was associated with bleeding. Despite bleeding being the cause of death in some patients, bleeding was not associated with time to death after adjustments for known risk factors. The patients were transfused with a large amount of blood products compared to haematological non-ICU patients [9, 10]. This is not surprising, given the inherent bone marrow failure of either the disease itself or its treatment with chemotherapy, which 66% of patients had received less than 6 weeks prior to ICU admission. Thrombotic events also occurred in some patients despite low platelet counts.

It is well known that patients with haematological cancer receiving chemotherapy or undergoing HSCT have an increased risk of bleeding. The number of bleeding events has been studied as primary end-point in several large, multicentre platelet transfusion trials performed outside the ICU. In the PLADO (Platelet Dose) trial, including 1272 patients undergoing chemotherapy or HSCT, 67% had a bleeding episode before leaving the hospital or within 30 days [9]. In the TOPPS study (Trial of Prophylactic Platelets), including 598 patients undergoing chemotherapy for haematological cancer or HSCT, 47% of the 598 patients bleed within 30 days [10]. Both studies had low platelet levels as one of the inclusion criteria.

Likewise, in a German platelet dose study, 391 patients undergoing HSCT or intensive chemotherapy for AML were randomised to receive platelets either when bleeding or when platelet counts were less than 10 × 10^9^ per litre. Patients were only followed as long as platelet counts were less than 20 × 10^9^ per litre. In the HSCT group, grade 3 bleedings were rare and grade 4 bleedings non-existent. In the leukaemia group, 15 patients had non-fatal haemorrhages, including six with minor cerebral bleeds. Two fatal intracranial bleeds were registered, both in the group receiving platelets when bleeding [16].

Our study population was small compared to the three trials described above, but overall we found the same number of bleeding complications. In other words, despite our patients having considerably shorter observation periods the same incidence of bleeding was found. We also found that our patients had longer bleeding episodes. The median number of days with bleeding in the PLADO study was one day, whereas our patients bled for 3 days. Importantly, in our study population, more than half of the bleedings were severe or debilitating and 8% had fatal bleedings. In contrast, in PLADO 10% of the patients had WHO grade 3 or 4 bleedings and only one patient died from bleeding. In TOPPS, 1% (7 patients) had grade 3 or 4 bleeding episodes and none died from bleeding.

Patients with lower platelet levels did have a higher frequency of bleeding complications. Most patients received prophylactic platelet transfusions when platelet levels fell below 20 × 10^9^ per litre, although the platelet levels at which the individual patient was transfused was at the clinicians’ discretion. The proportion of severe and debilitating bleedings (WHO grade 3–4) did not decrease as might have been expected at increasing platelet levels, which probably explains why platelet levels did not influence survival in our population. However, use of platelet and RBC transfusions was increasing in the days leading up to a severe bleeding, which might indicate that increasing platelet dependency and minor bleedings could be warning signs of severe/debilitating bleeding.

We also analysed biochemical coagulation parameters and calculated the disseminated intravascular coagulation (DIC) scores according to the definition by the International Society of Thrombosis and Haemostasis (ISTH) [25]. Among these other markers of coagulation (International normalised ratio (INR), activated pro-thrombin time (APTT), anti-thrombin, D-dimer and fibrinogen) only INR was weakly associated with subsequent bleeding (odds ratio 2.91 for bleeding within 24 h, 95% CI 1.01–8.43, *p* = 0.048). The DIC score was neither associated with bleeding nor thrombosis. None of the biochemical parameters had any predictive value with regard to bleeding in these patients as described elsewhere [26].

The majority of bleedings in our ICU started within the first 5 days. Several bleedings were severe and in nine patients (8%) the direct cause of death, thereby contributing to the high mortality. More than half of the patients died in ICU, and the 30-day mortality was 60%. This is similar to the mortality rates described by Pène et al. in critically ill HSCT patients [27], but higher than described in some other studies of critically ill haematology patients. However, our population differs from some of the previous studies [3, 5, 28–31] in that the vast majority were mechanically ventilated (87%) and received vasopressor (82%). In most other studies, the ratio of mechanically ventilated patients has been considerably lower, in some less than 50% [3, 30–32].

So why are our patients bleeding more? Sepsis-induced coagulopathy causes bleeding in ICU patients [33], so the risk of bleeding is likely to be increased in leukaemia patients with sepsis and a large proportion of our patients indeed had sepsis at ICU admission. A low platelet count has been associated with adverse outcome in a general population of ICU patients [32, 34–37] although thrombocytopenia as a determinant of severe bleeding in ICU remains unclear. Several studies have assessed the risk of bleeding in ICU patients with thrombocytopenia [34, 37–39], and most have found a higher risk of bleeding in patients with lower platelet counts [36, 38, 40] with the exception of one study, which found that sepsis was the single variable associated with bleeding in multivariate analysis [41]. However, none of these studies were in haematological patients only.

Although the incidence of bleeding in medical ICU patients without haematological malignancies is lower, the exact numbers are not certain, as haematological patients often are included in the general ICU populations. The prospective multicentre Scandinavian Starch for Severe Sepsis/Septic Shock (6S) trial [42], which included 798 general ICU patients with severe sepsis, had severe bleeding (defined as clinical bleeding that required 3 or more units of packed red cells within 24 h) as one of the pre-defined outcomes. In 6S, 19% of the patients had a bleeding episode and 8% had severe bleeding [43]. However, 35% of the patients had surgery prior to ICU admission. Among the patients without prior surgery, only 16% had a bleeding episode and 6% had severe bleeding. Haematological patients were included in this study, constituting 9% of the total study population.

Some of our patients also had thrombotic events in spite of low platelet counts, which was the cause of death in four patients. The patients with thrombosis also had low platelet counts on the day of the thrombus, and several of them had one or more bleeding episodes before the thrombotic episode. All except one of the thrombotic events in our study were in patients with AML. Patients with acute leukaemia have an increased risk of thrombosis that may be as high, or even higher than in patients with solid tumours [44, 45]. There are several reasons why thrombocytopenic leukaemia patients still have an increased risk of thrombosis [8, 46, 47]:  Leukaemic cells express pro-coagulant mediators including tissue factor [48, 49], produce pro-inflammatory and pro-coagulant cytokines [50, 51], release leukaemia cell derived microparticles into the blood stream which are expressing tissue factor on the surface [52] and directly activate platelets [53, 54]. The rapid cell death induced by chemotherapy further increases the release of pro-coagulant factors into the blood stream [50]. Furthermore, all our patients had central venous catheters which is a likely risk factor for thrombosis in patients with acute leukaemia, although the incidence has varied in different studies and no studies have been in ICU patients only [55–57].

Preventing thromboembolic complications in thrombocytopenic patients with haematological malignancy is indeed complicated due to the high risk of bleeding. There are no available guidelines for prevention of thrombosis in acute leukaemia patients outside the ICU setting, and in ICU the risk is likely to be even more increased due to immobilisation, use of vasopressors and sepsis [58, 59]. To our knowledge, no randomised trials on thrombosis prophylaxis in leukaemia patients have been made. A prospective multicentre study on the incidence of thrombosis in non-ICU haematological patients with central venous catheters found no increase in bleeding in the 14% of patients receiving thrombosis prophylaxis [55]. However, even though thrombosis prophylaxis was not found to increase the number of bleeding episodes, the incidence of thrombosis was not reduced. Similar results were found in a retrospective study of central venous catheters in AML patients [57] as well as in a study of low-dose warfarin prophylaxis in oncology patients [60]. In our study, 47 patients (41%) received enoxaparin as thrombosis prophylaxis during the ICU stay. No increase in bleeding events was observed among these patients.

Our patients received large amounts of blood products. Severely thrombocytopenic patients would be expected to bleed more and therefore receive more RBC transfusions, but this was not the case. Bleeding patients did, for obvious reasons, receive more blood products than non-bleeding patients, but it is worth noticing that 36% of the patients who received RBC transfusion and 27% of the patients who received fresh frozen plasma did not have any signs of clinical bleeding.

This study has several limitations. Due to its retrospective nature, there is a risk of underestimating the occurrence of minor bleedings. Furthermore, this is a single-centre study and it may be that the results are different in other centres. Most importantly, we cannot make any inferences about cause and effect because of the observational design, in particular as the time at risk of bleeding for any patient is related to the time spent in ICU, and as such it is difficult to analyse any associations to mortality.

The strength of this study is the high availability of data achieved by using three separate databases linked using the patients’ unique identification number. We also included a relatively homogenous group of patients with only three acute haematological diagnoses, and where the majority had sepsis, required mechanical ventilation and had received chemotherapy within 6 weeks of ICU admission. We had few exclusions and full follow-up of all included patients.

## Conclusions

ICU patients with acute leukaemia and MDS had a high risk of severe, debilitating and fatal bleeding episodes in the ICU and lower platelet counts appeared to be a risk factor for bleeding. Preventing severe bleeding episodes appears to be crucial, and close daily monitoring of minor bleedings and increasing platelet dependency might aid this goal. Further research is warranted in order to avoid debilitating and fatal bleeding without increasing the risk of thrombosis. It seems worthwhile to test the benefits and harms of therapeutic interventions, e.g. higher platelet trigger values in high-risk patients and alternatives to platelet transfusions to prevent bleeding as well as the benefits/risk of thrombosis prophylaxis in thrombocytopenic ICU patients with acute leukaemia and MDS.

## Additional file

**Additional file 1: Table S1.** Grading of bleeding according to the World Health Organisation.

## Abbreviations

ALL: acute lymphoblastic leukaemia
AML: acute myeloid leukaemia
CI: confidence interval
CT: computered tomography
FFP: fresh frozen plasma
GI: gastrointestinal
HSCT: haematopoietic stem cell transplantation
ICH: intra-cranial haemorrhage
ICU: intensive care unit
IQR: interquartile range
LMWH: low molecular weight heparin
MDS: myelodysplastic syndrome
OR: odds ratio
PLADO: platelet dose study
RBC: red blood cell
SAPS: Simplified Acute Physiology Score
SOFA: Sequential Organ Failure Assessment
TOPPS: trial of prophylactic platelets
WBC: white blood cell
WHO: World Health Organisation
