# Supplementary material for: Risks of bleeding and thrombosis in intensive care unit patients with haematological malignancies
Source: Ann Intensive Care. 2017 Dec 11;7:119. doi: 10.1186/s13613-017-0341-y (PMC5725397; doi:10.1186/s13613-017-0341-y)
Supplement: Supplementary file 1 — Additional file 1: Table S1. Grading of bleeding according to the World Health Organisation. [file 13613_2017_341_MOESM1_ESM.docx]

**SUPPLEMENTARY MATERIAL**

|  | |
| --- | --- |
| Table S1. Grading of bleeding according to the World Health Organisation ^a)^ | |
| Grade 1: Minor Blood Loss | |
|  | Petechiae, oropharyngeal bleeding |
|  | Epistaxis (< 1 hour) |
|  | Purpura (< 2.5 cm diameter) |
|  | Stool occult blood (trace to 1+) |
|  | Urine haemoglobin (trace to 1+) |
|  | Subconjunctival bleeding |
|  | Abnormal vaginal bleeding (non-menstrual) |
| Grade 2: Mild Blood Loss | |
|  | Melena, hematemesis, haemoptysis, haematuria, haematochezia and abnormal vaginal bleeding NOT requiring RBC transfusion |
|  | Retinal bleeding without visual impairment |
|  | Epistaxis or oropharyngeal bleeding >1 hour |
|  | Stool occult blood (moderate or 2+ and greater) |
|  | Urine haemoglobin (moderate or 2+ and greater) |
|  | CNS bleeding noted on CT without clinical consequences |
| Grade 3: Severe Blood Loss | |
|  | Melena, hematemesis, haemoptysis, haematuria, haematochezia, abnormal vaginal bleeding, epistaxis and oropharyngeal bleeding and bleeding from invasive site, requiring RBC transfusion |
| Grade 4: Debilitating Blood Loss ^b)^ | |
|  | Debilitating bleeding including retinal bleeding with visual impairment, non-fatal bleeding with neurological signs and symptoms, bleeding associated with hemodynamic instability and fatal bleeding from any source |

Abbreviations: ***CNS*:** central nervous system; **RBC**: red blood cells.

^a)^ We used the specific descriptors for each grade of bleeding as previously described by Heddle et al. Blood 2009^20^

b) We included ‘Bleedings associated with haemodynamic instability’ as a grade 4 bleeding as described by Stanworth et al. NEJM 2013^10^
